# *Lactobacillus plantarum* favors the early emergence of fit and fertile adult *Drosophila* upon chronic undernutrition

**DOI:** 10.1242/jeb.151522

**Published:** 2017-03-01

**Authors:** Mélisandre A. Téfit, François Leulier

**Affiliations:** Institut de Génomique Fonctionnelle de Lyon, Université de Lyon, Ecole Normale Supérieure de Lyon, Centre National de la Recherche Scientifique, Université Claude Bernard Lyon 1, Unité Mixte de Recherche 5242, Lyon, Cedex 07 69364, France

**Keywords:** Microbiota, Symbiosis, Fertility, Fitness, Lifespan

## Abstract

Animals are naturally surrounded by a variety of microorganisms with which they constantly interact. Among these microbes, some live in close association with a host and form its microbiota. These communities are being extensively studied, owing to their contributions to shaping various aspects of animal physiology. One of these commensal species, *Lactobacillus plantarum*, and in particular the *L.p.^WJL^* strain, has been shown to promote the growth of *Drosophila* larvae upon nutrient scarcity, allowing earlier metamorphosis and adult emergence compared with axenic individuals. As for many insects, conditions surrounding the post-embryonic development dictate key adult life history traits in *Drosophila*, and adjusting developmental timing according to the environment is essential for adult fitness. Thus, we wondered whether the growth acceleration induced by *L.p.^WJL^* in a context of poor nutrition could adversely impact the fitness of *Drosophila* adults. Here, we show that the *L.p.^WJL^*-mediated acceleration of growth is not deleterious; adults emerging after an accelerated development are as fit as their axenic siblings. Additionally, the presence of *L.p.^WJL^* even leads to a lifespan extension in nutritionally challenged males. These results demonstrate that *L.p.^WJL^* is a beneficial partner for *Drosophila melanogaster* through its entire life cycle. Thus, commensal bacteria allow the earlier emergence and longer survival of fit and fertile individuals and might represent one of the factors contributing to the ecological success of *Drosophila*.

## Abstract (precis)

**Summary:** *Lactobacillus plantarum^WJL^* is beneficial to *Drosophila* physiology across its entire life cycle, triggering the early emergence and longer survival of fit and fertile adults.

## INTRODUCTION

In nature, animals are constantly surrounded by a profusion of microorganisms, whose presence has contributed to shaping life as we know it (McFall-Ngai, 2015). The interactions existing between microbes and animals cover a broad spectrum, with outcomes ranging from obligate symbiosis to lethal infection (Casadevall and Pirofski, 2000; Hentschel et al., 2000). Among these microbial species, some live in close association with an animal host with which they establish commensalistic or mutualistic relationships. The community they form is referred to as the microbiota, which over recent years has been increasingly studied for its impact on various physiological traits. Indeed, in several mammalian, nematode or arthropod models, the microbiota has been shown to shape development, immunity, metabolism and even behavior (Kostic et al., 2013; Lee and Hase, 2014). In this fast expanding research field, *Drosophila* has been a fruitful model. Thanks to its ease of manipulation and genetic tractability, as well as the low complexity of its microbiota, the fruit fly represents a powerful tool to delve into the mechanistic underpinnings of host–microbiota interactions (Lee and Brey, 2012; Ma et al., 2015). Studies have revealed that the presence and composition of microbiota impact various traits throughout the *Drosophila* life cycle such as larval growth, developmental timing, stress resistance, immune response, metabolism, lifespan and behavior (Brummel et al., 2004; Ryu et al., 2008; Sharon et al., 2010; Shin et al., 2011; Guo et al., 2014; Petkau et al., 2014; Venu et al., 2014; Wong et al., 2014; Clark et al., 2015). As the microbiota is closely associated with its animal partner and, in the case of *Drosophila*, is an integral part of its nutritive substrate, it is not surprising to see its influence on so many biological functions. Moreover, as for many insects, the larval stage is highly plastic in the fly life cycle. Indeed, biotic and abiotic factors surrounding the development of an organism participate in shaping this process (Gilbert, 2001; McFall-Ngai, 2002), and in turn have a crucial impact on several key life history traits at the adult stage, such as reproductive capacity, stress resistance or lifespan (Tu and Tatar, 2003; Andersen et al., 2010; Sisodia and Singh, 2012; Burns et al., 2012).

Previously, we showed that, upon mono-association, some strains of the commensal bacterial species *Lactobacillus plantarum* (a member of the dominant phyla of *Drosophila* microbiota) are able to sustain the systemic growth of *Drosophila* larvae to the same extent as a more complex microbiota (Storelli et al., 2011; Erkosar et al., 2015). Upon yeast deprivation during the larval stages, mono-association of germ-free animals with the strain *Lactobacillus plantarum^WJL^* (*L.p.^WJL^*) isolated from the intestine of lab-raised *Drosophila melanogaster* (Ryu et al., 2008) increases larval growth and reduces developmental timing, thus allowing earlier entry into metamorphosis of mono-associated individuals (Storelli et al., 2011).

Several studies using *Drosophila* lines generated in a laboratory evolution experiment of postponed senescence selection (Rose, 1984) have described a series of trade-offs between key life history traits. This occurs when the optimization of a trait correlates with a negative impact on another parameter; for example, increased reproductive capacity usually comes at the cost of a shortened lifespan. Such trade-offs can involve traits either from the same life stage or across different life stages, and thus the length of the larval period, early and late life fecundity, adult longevity and stress resistance were shown to trade-off with one another (reviewed in Zera and Harshman, 2001). Given the numerous examples of life history trade-offs and the rather striking effect of *L.p.^WJL^* on larval development, we wondered about the potential repercussions of this accelerated growth on adult fitness. We speculated that *L.p.^WJL^*-mediated acceleration of growth in an otherwise nutritionally challenging environment might be deleterious at later stages such that it would lead to the emergence of unfit adults. To address this question, we assessed several fitness parameters in young adult flies and observed that, overall, *L.p.^WJL^* association was not detrimental for adult fitness. Furthermore, for adult males it proved to be an advantageous partner; *L.p.^WJL^*-associated males not only emerged several days before their germ-free siblings but also survived longer in nutritionally challenging conditions. *L.p.^WJL^* is thus a true beneficial partner for *Drosophila* across its entire life cycle, and even more so in a poor nutritional environment. This study therefore supports the notion that bacterial members of the fly microbiota might represent one of the factors contributing to the ecological success of *Drosophila melanogaster*.

## MATERIALS AND METHODS

### Fly stocks and husbandry

*yw* fly stocks were reared on a standard yeast/cornmeal diet containing (for 1 l): 50 g inactivated yeast (Bio Springer, Springaline BA95/0-PW), 80 g cornmeal (Westhove, Farigel maize H1), 10 g agar (VWR, ref. 20768.361), 5.2 g methylparaben sodium salt (Merck, ref. 106756) and 4 ml 99% propionic acid (Carlo Erba Reagents, ref. 409553). All experimental flies were kept in incubators at 25°C, with a 12 h:12 h light:dark cycle. The low-yeast diets were made by decreasing the quantity of yeast to 30, 12, 8 or 6 g l^−1^ and the quantity of agar to 7.2 g l^−1^. Unless stated otherwise, only mated flies were used in this study.

### Generation of axenic *Drosophila* stocks and bacterial mono-association

To generate axenic flies, eggs were collected overnight and treated in sterile conditions with successive 2 min baths of bleach and 70% ethanol. Bleached embryos were then rinsed in sterile water for another 2 min and placed on sterile standard food supplemented with an antibiotic cocktail (50 μg ampicillin, 50 μg kanamycin, 50 μg tetracycline and 15 μg erythromycin per liter of fly food). Emerging adults were tested for axenicity by crushing and plating of the fly lysate on different bacterial culture media. The presence of tetracycline in the antibiotic cocktail ensured the absence of *Wolbachia* in the axenic stocks. Germ-free flies were kept on antibiotic food for a few generations and conventionally reared stocks were used to regenerate axenic stocks regularly. For bacterial mono-association, 50 μl of PBS containing 10^8^ CFU of a stationary phase culture of *L.p.^WJL^* was used to inoculate the surface of antibiotic-free fly food contained in a 1.5 cm diameter fly tube. Fifty axenic eggs from an overnight collection were transferred onto the inoculated food and left to develop until adult emergence. The experimental germ-free condition was obtained by inoculating the food with sterile PBS. For association at the adult stage, antibiotic-free fly food was inoculated as described above and left to dry under a hood; 40–50 newly emerged adult flies (females and males mixed 1:1) were then transferred into the inoculated tubes and reared for 7 days until the beginning of the experiments.

### Developmental timing

Fifty germ-free embryos were associated with *L.p.^WJL^* or kept axenic, as described above. Larvae were then left to develop under low nutrient conditions (low-yeast diet, 8 g l^−1^ yeast) and the number of pupae appearing each day was recorded until the last larvae of the population reached pupariation.

### Fecundity and fertility assessment

At emergence, groups of five females and five males were distributed in vials and transferred every 24 h to a new tube. The number of eggs laid was recorded every day for 10 days and the subsequent number of emerging adults was used to calculate the fertility ratio (number of emerging progeny divided by the number of eggs laid). In experiments where bacterial association was done only at the adult stage, the fecundity/fertility assays were started at day 7 or 10 after adult emergence and continued for 3–7 days.

### Number of ovarioles

Mated females, 4–5 days old, were used to assess the number of ovarioles after development on either standard (50 g l^−1^ yeast) or low-yeast (8 g l^−1^ yeast) diet. Newly emerged adult flies were kept on standard food until the time of dissection. Ovaries were dissected in cold PBS and directly fixed in 4% formaldehyde for 20 min. They were then stained with DAPI (1:1000) for 15 min and transferred to 80% glycerol for preservation. After fixation and staining, ovarioles were teased apart under a dissecting microscope and mounted on slides for counting.

### Adult wet mass and resistance to full starvation

Either virgin (0–7 h old) or mature and mated adults (7 or 10 days old) were collected and pooled in groups of five to be weighed on a Sartorius analytical balance CPA324S (Sartorius Weighing Technology GmbH, Goettingen, Germany). Flies of the same age were also used for full starvation assays, in tubes providing only water supply to the flies. Specifically, the starvation tubes contain a cotton ball soaked in a water reservoir to prevent them from drying. The cotton is covered with a piece of Whatmann paper on which the flies are placed. Survival of the flies was recorded twice a day until all individuals were dead.

### Lifespan

After larval development on either standard (50 g l^−1^ yeast) or low-yeast (8 g l^−1^ yeast) diet, newly emerged adults were kept all together for 3–4 days before males and females were separated for the subsequent experiments. Groups of 10 mated flies were transferred to fresh vials containing either standard or low-yeast diet. Flies were transferred to fresh fly food tubes twice a week and survival was recorded daily until all individuals were dead. Depending on the condition and on the experiment, 5–10 replicates were performed.

### Statistical analyses

For comparison of GF and *L.p.^WJL^*-associated conditions, Mann–Whitney test (for mass, fecundity, fertility) and logrank test (for survival curve comparison) were performed using GraphPad Prism software version 6.0f for Macintosh (GraphPad Software, La Jolla, CA, USA; www.graphpad.com). The results of the Brown–Forsythe test for comparison of standard deviations were also obtained with this software. Whiskers of the boxplots represent the minimal to maximal values. For all experiments, the *P*-values are reported on the corresponding figure panels only when <0.05.

## RESULTS

### *L.p.^WJL^* does not directly impact *Drosophila* adult fitness

**Fig. 1.**
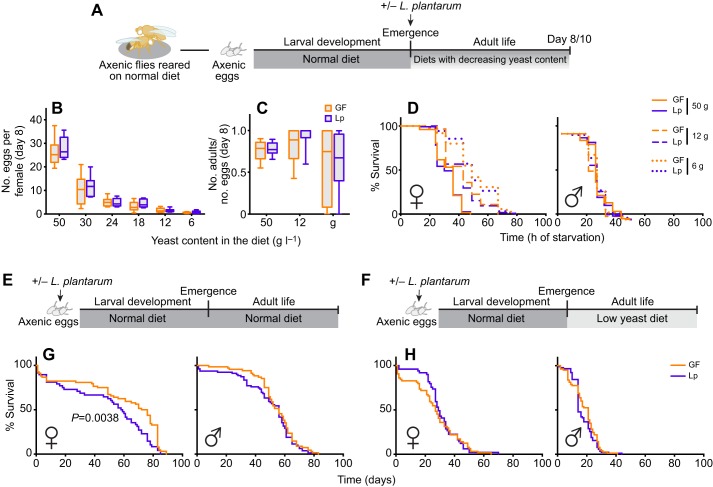
***Lactobacillus plantarum^WJL^* (*L.p.^WJL^*) does not directly impact *Drosophila* adult fitness.** (A) Immediately after emergence, axenic (germ-free, GF; devoid of microbiota) adults developed on a normal diet were associated with *L.p.^WJL^* (Lp) or sterile PBS. (B–D) When mature, they were tested for fecundity (B) or fertility (C) at 8 days of age, and for resistance to complete starvation (D) at 10 days after emergence. (E–H) Axenic eggs were inoculated with *L.p.^WJL^* or sterile PBS and developed on a normal diet (50 g l^−1^). The lifespan of the adults was then assessed on either the same normal diet (E,G) or a diet with a reduced amount of yeast (8 g l^−1^; F,H).

To determine whether *L.p.^WJL^* had an impact on fly physiology at the adult stage, we first assessed the direct effect of *L.p.^WJL^* on adult *Drosophila*, by associating newly emerged flies with the bacteria. After larval development on a normal diet in axenic conditions (germ-free, GF; devoid of microbiota), the young emerging adults were either associated with *L.p.^WJL^* or kept axenic (Fig. 1A). The flies were left to mature for several days on diets with decreasing amounts of yeast and were then tested for fecundity, fertility and resistance to full starvation. After 8 days in various nutritive conditions, there was a clear effect of diet composition on the number of eggs laid per female and on the number of adult progeny emerging from these eggs; with decreasing amounts of yeast in the diet, the flies laid fewer eggs (Fig. 1B) and the fertility ratio (number of emerging progeny/number of eggs laid) showed a statistically significant increase in variability (Fig. 1C; Table S1). The ability of females to endure complete starvation was also impacted by the amount of yeast in the diet. Indeed, 7 day old females survived longer when they had been kept on a low-yeast diet after emergence (Fig. 1D, left panel). In contrast, the diet composition did not matter for their male counterparts, which died at the same rate regardless of the diet they were kept on after emergence (Fig. 1D, right panel). The association with *L.p.^WJL^*, however, did not impact any of these adult fitness traits. In addition, we tested the same parameters in flies that were raised on a normal diet in the presence of *L.p.^WJL^* during larval life. In such rich nutritional conditions, the developmental time was similar for the axenic and the *L.p.^WJL^*-associated flies, and here again there was a clear impact of diet composition on fecundity, but no bacterial contribution was revealed for either fecundity or resistance to full starvation (Fig. S1). We next assayed the lifespan of these flies raised with or without *L.p.^WJL^* on a normal diet, and kept as adults on either the same rich diet or a low-yeast food (Fig. 1E,F). Here, we saw a significant increase in the lifespan of axenic females kept in nutritionally rich conditions throughout their life cycle (Fig. 1G, left panel). For their male counterparts, however, as well as for female and male flies that went from a larval development on a normal diet to adult life on a low-yeast diet, there was no significant impact of *L.p.^WJL^* presence (Fig. 1G, right panel, and 1H). Taken together, these results show that apart from the previously described sexually dimorphic lifespan shift on a normal diet (i.e. increased lifespan in GF females; Petkau et al., 2014; Clark et al., 2015), association of *Drosophila* with *L.p.^WJL^* does not seem to have a direct impact on adult fitness when flies develop on a normal diet.

### *L.p.^WJL^*-mediated larval growth acceleration is not deleterious for adult fitness

**Fig. 2.**
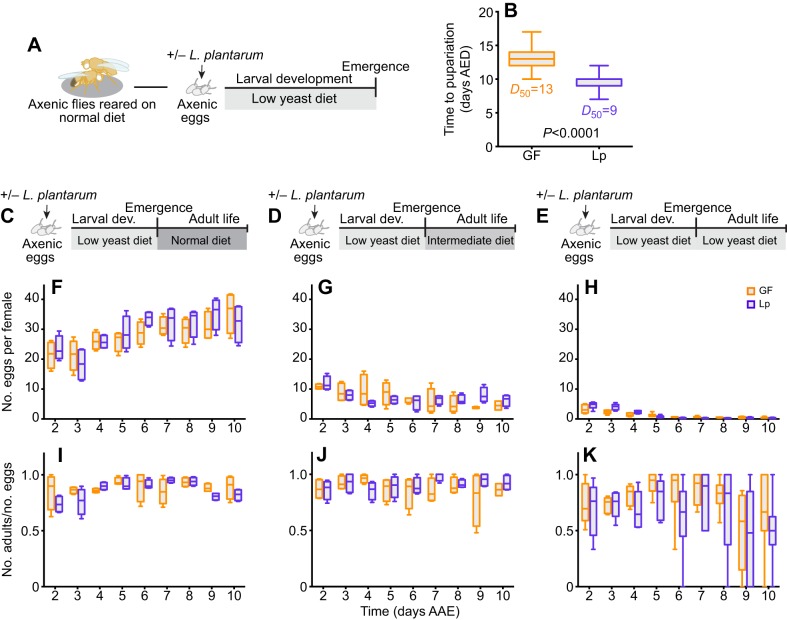
***Drosophila* reproductive capacity is not altered after an accelerated larval development.** For larvae raised on a low-yeast diet (8 g l^−1^; A), the presence of *L.p.^WJL^* accelerated development and shortened the time to pupariation (B; AED, after egg deposition). The *D*_50_ values represent the time (days) that 50% of the population reached pupariation. After this differential development, adults were kept on a rich diet (50 g yeast l^−1^; C), an intermediate diet (30 g yeast l^−1^; D) or a nutritionally poor diet (8 g yeast l^−1^; E) and assessed for fecundity and fertility from day 2 after adult emergence (AAE) to day 10. The corresponding number of eggs laid per female per day (F–H) and the fertility ratios (emerging adult progeny divided by the number of eggs laid; I–K) are shown.

**Fig. 3.**
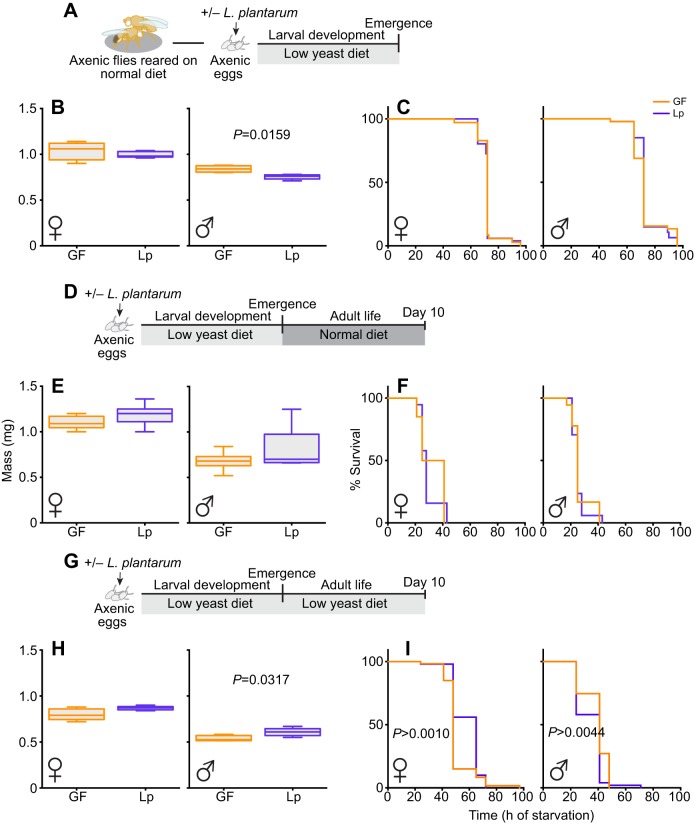
***L.p.^WJL^*-mediated larval growth acceleration is not deleterious for adult fitness.** (A) Larvae were developed on a low-yeast diet. Immediately after emergence (0–7 h old), fly mass (B) and resistance to full starvation (C) were assessed. The same parameters were then tested on 10 day-old adults kept on either a normal diet (D–F) or a low-yeast diet (G–I).

While searching for a direct effect of *L.p.^WJL^* on the adult stage, we did not detect any significant impact of the commensal bacteria on the tested fitness parameters. There was, however, a quite striking larval effect, as nutritionally challenged individuals developed faster and pupariated several days earlier when they were associated with *L.p.^WJL^* compared with the axenic ones (Storelli et al., 2011; Erkosar et al., 2015). While faster larval growth and precocious emergence of the adult represent an obvious ecological advantage, doing so under nutritionally challenging conditions may in turn be deleterious for adult fitness and reproductive success. Indeed, adjusting developmental timing to environmental cues is key to *Drosophila* adult fitness (Nylin and Gotthard, 1998), yet upon *L.p.^WJL^* association animals develop faster even though the nutritional conditions are poor. To investigate whether the growth acceleration mediated by *L.p.^WJL^* upon nutrient scarcity would adversely impact subsequent adult fitness, we tested flies raised on a low-yeast diet with or without the bacteria, as depicted in Fig. 2A. As previously described, when raised on a low-yeast diet, larvae associated with *L.p.^WJL^* pupariate several days before their axenic siblings (Storelli et al., 2011; Erkosar et al., 2015; Fig. 2B). We then assessed the potential repercussions of the *L.p.^WJL^* association on the reproductive capacity of flies that underwent larval development in such nutritionally challenging conditions. Similar to what we observed when the flies were grown in nutrient-rich conditions and challenged only as adults, fecundity (Fig. 2F–H) and fertility (Fig. 2I–K) were both greatly impacted by adult diet composition (Fig. 2C–E). The higher the yeast content in the diet, the more eggs were laid per female per day (Fig. 2F–H). In addition, the number of adult progeny emerging from these eggs was impaired on the low-yeast diet. Indeed, as we observed for the low-yeast diet in Fig. 1C (6 g l^−1^ of yeast), on the 8 g l^−1^ yeast diet the fertility ratio was very variable (Fig. 2I–K; Table S1). For these two parameters, again, there was no impact of the association with *L.p.^WJL^*. Furthermore, these comparable fecundity results were supported by the fact that the number of ovarioles (the functional units of *Drosophila* ovaries) of females raised on a low-yeast diet was similar, regardless of their microbiota status (Fig. S2A). We are confident that our experimental setup can efficiently manipulate the ovariole number because, as expected, we observed a decreased count after development on a low-yeast diet compared with a nutritionally rich situation (Fig. S2A; Hodin and Riddiford, 2000; Tu and Tatar, 2003). As anticipated, the similar number of ovarioles between the GF and *L.p.^WJL^* conditions translated into a similar cumulative number of eggs laid over the course of the experiment, and as expected we detected reduced cumulative egg laying when animals developed on the poor diet (Fig. S2B). Next, we assayed the mass of 0–7 h old virgin adults, along with their resistance to complete starvation, as indicators of direct consequences of larval life on their adult metabolic state (Baker and Thummel, 2007). We detected a slight tendency in males and females associated with *L.p.^WJL^* to weigh less than axenic ones (Fig. 3A,B), but there was no impact of the growth acceleration mediated by *L.p.^WJL^* on the flies' ability to endure full starvation (Fig. 3C). These assays were repeated on mature adults, after 10 days of adult life on either a normal diet (Fig. 3D–F) or the same low-yeast diet (Fig. 3G–I) and, again, there was no deleterious impact of the *L.p.^WJL^*-mediated growth promotion on these adult fitness parameters. At this age, the mass tendency was reversed, as *L.p.^WJL^*-associated males and females were now slightly heavier than their axenic counterparts. Similarly to what we observed with newly emerged flies, this did not translate into differences in resistance to full starvation. In addition, similar results were obtained when we starved adult flies that were matured on a diet with an intermediate yeast content (Fig. S3). Notably, for some of these experiments, the statistical analyses show significant differences between the groups, but the differences they represent are tenuous and probably not of any biological relevance. Collectively, these data suggest that even though larvae associated with *L.p.^WJL^* develop faster in an otherwise poor nutritive environment, they do so without generating fitness costs for the later stage and give rise to fit and fertile adults.

### *L.p.^WJL^* increases the lifespan of nutritionally challenged males

**Fig. 4.**
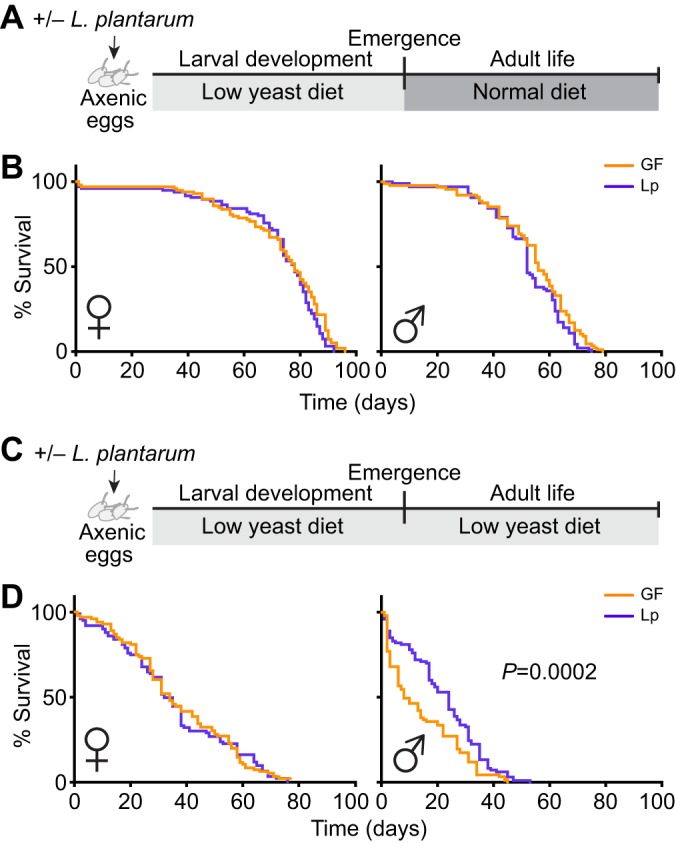
***L.p.^WJL^* increases the lifespan of nutritionally challenged males.** The lifespan of adult males and females was assessed after larval development under low nutrition with or without *L.p.^WJL^*, when flies were kept on either a normal diet (A,B) or the same low-yeast diet as the larvae (C,D).

While performing the experiments, we noticed that when kept on a low-yeast diet, adult males were dying rapidly and a significant proportion of them were dead 10 days after emergence. We then decided to study in more detail the lifespan of flies raised in such nutritionally poor conditions. After emergence from larval development on a low-yeast diet, the adults were either kept on the same low-yeast diet (Fig. 4C) or transferred to a rich diet (Fig. 4A). We saw that, while the larval nutritional environment had a notable impact on female lifespan, with a poor larval diet translating into an increased female lifespan (Fig. S4A), association with *L.p.^WJL^* did not impact this trait (Fig. 4B). However, males maintained on a low-yeast diet throughout their entire life survived better when they were associated with *L.p.^WJL^* (Fig. 4D, right panel; Fig. S4B). Notably, their median lifespan was extended by 4–16 days, depending on the experiment. This fluctuation in the actual day count across experiments is commonly seen in lifespan studies (Piper et al., 2013) but the trend persisted and was statistically significant. This result shows that in a nutritionally challenging environment, *L.p.^WJL^* association not only shortens *Drosophila* developmental time but also significantly increases the lifespan of adult males.

## DISCUSSION

The microbiota is one of the key environmental factors impacting animal development and physiology and has been increasingly studied over the last few years (Sommer and Bäckhed, 2013). Our work focuses on the association between *Drosophila melanogaster* and one of its natural commensal partners, *L.p.^WJL^*. The findings of this study broaden our understanding of the relationship between these two partners and show that *L.p.^WJL^* is beneficial for the fly throughout all life stages. We first tested whether *L.p.^WJL^* had a direct impact on adult fitness traits, by associating newly emerged flies following larval development in axenic conditions. We showed that, in our setup, bacterial presence is dispensable for adult fitness; the flies laid the same number of eggs and resisted starvation equally well, whether they were mono-associated or not. Notably, and contrary to the effect of *L.p.^WJL^*, the composition of the diet markedly impacted these parameters, and we observed a negative correlation between starvation resistance and egg laying. When decreasing the amount of yeast in the diet, we saw an extension of survival upon complete starvation for female flies, together with a drop in the number of eggs laid. This effect was not only microbiota independent but also sex specific and the starvation resistance of males was not impacted by the quantity of yeast in the diet. Similar observations were previously reported in a study where starvation resistance was promoted by lower yeast levels in the diet, at the expense of fecundity (Chippindale et al., 1993). As in the present study, this effect was restricted to females, a characteristic that the authors attributed to distinct lipid requirements between the sexes.

In a previous study, we compared the transcriptomes of germ-free versus poly-associated flies that had been inoculated at adult stage with a cocktail of four bacterial species selected to represent the main commensals of *Drosophila* (*Acetobacter pomorum*, *Commensalibacter intestini*, *Lactobacillus brevis* and *Lactobacillus plantarum*) (Erkosar et al., 2014). This analysis revealed a differential expression of several genes pertaining to metabolic processes; out of 105 transcripts upregulated upon bacterial poly-association, 74 were metabolism related. With such a differential expression of metabolic genes, one could expect that certain fitness parameters, like reproductive capacity or starvation resistance, would be affected. The present study revealed no differences between germ-free and *L.p.^WJL^*-associated animals for these traits. Given the link between metabolism and adult locomotor capacity, it would now be interesting to investigate the activity of germ-free versus microbe-associated flies.

The differences between our previous transcriptomic analysis and our phenotypical analysis might be attributed to the association set-up. In this study, the flies were mono-associated with one species of *Lactobacillus* while in Erkosar et al. (2014) the animals were poly-associated. Moreover, the bacterial cocktail used in Erkosar et al. (2014) contained a species belonging to the *Acetobacter* genus. Lactobacilliaceae and Acetobacteraceae are the most represented bacterial families in the communities associated with *Drosophila* populations, in laboratory stocks as well as in wild-caught flies (Broderick and Lemaitre, 2012; Staubach et al., 2013; Chaston et al., 2016). Several studies have shown the impact of *Acetobacter* species, notably *A. pomorum* and *A. tropicalis*, on the metabolism of adult *Drosophila*, both upon mono-association with one species and in bacterial mixtures (Newell and Douglas, 2014; Huang and Douglas, 2015; Chaston et al., 2016; Elgart et al., 2016). Furthermore, these studies specifically addressed the differential impact of *Acetobacter* species versus *Lactobacillus* species and demonstrated that, in their setup, the latter had little to no effect in comparison to the former (Newell and Douglas, 2014; Huang and Douglas, 2015; Chaston et al., 2016; Elgart et al., 2016). These observations may be due to the intrinsic differences in the metabolic end-points of these bacteria. Indeed, while *Acetobacter* spp. are obligatory aerobes, nitrogen-fixing and acetic acid-producing bacteria, most *Lactobacillus* spp. are facultative anaerobes and heterofermentative microorganisms; they use sugars and pyruvate-derived carbon, and produce either alcohol or lactic acid. It must be pointed out, however, that beyond the distinction between bacterial species, the strain considered is important. Indeed, our lab and others have shown that various microbial effects are strain specific (Storelli et al., 2011; Chaston et al., 2014). Nevertheless, taken together, all these observations suggest that adult *Drosophila* fitness traits might be influenced by the presence of the Gram-negative, acetic acid-producing *Acetobacter* species rather than by the Gram-positive, lactic acid-producing *Lactobacillus* species*.* However, based on the potent influence on larval systemic growth of *Lactobacillus* strains, we suspect that adult ‘growth’-related traits such as tissue regeneration (the intestine in particular) might be impacted by *Lactobacillus*.

Having ruled out a direct impact of *L.p.^WJL^* on adult fitness, we wanted to investigate the potential repercussions of the bacteria-mediated larval growth acceleration on adult flies. When larvae are raised on a low-yeast diet, the presence of *L.p.^WJL^* promotes their growth and shortens their developmental timing (Storelli et al., 2011; Erkosar et al., 2015; this study). However, numerous studies have demonstrated that conditions impacting larval development are known to affect several adult traits in *Drosophila* and a shorter larval period could negatively trade-off with adult reproductive capacity, stress resistance or longevity (Zera and Harshman, 2001). We therefore suspected that this increased growth rate upon nutritional challenge could in turn adversely impact adult fitness. Here, we demonstrate that *L.p.^WJL^*-associated individuals are as fit as their GF siblings; they show similar reproductive capacity and resist complete starvation equally well, regardless of their developmental history. The association with *L.p.^WJL^* is thus overall profitable to the fly, as it promotes larval growth and the early emergence of the imago without impairing the fitness of this mature and reproductive stage.

Strikingly, we found that *L.p.^WJL^* extends the lifespan of males kept in poor nutritive conditions. Males that were kept on a low-yeast diet throughout their entire life cycle benefited from the bacterial presence both as larvae and as adults; they displayed a shortened developmental timing as well as an increased median lifespan compared with their GF siblings. Thus, *L.p.^WJL^*-associated males not only develop faster and emerge several days before their axenic counterparts but also survive longer. In the wild, where nutrients can be scarce, longer lifespan could grant these individuals more opportunities to mate, and to produce potentially more numerous progeny. However, to confirm this hypothesis, it is imperative to show that these early-emerged and long-lived males are superior in their healthspan. In this light, it might be of interest to assay the late-life reproductive capacity of *L.p.^WJL^*-associated versus GF flies to see whether, in addition to conferring the ability to live longer, *L.p.^WJL^* also allows males to stay fit and reproductively active longer. This is an interesting future direction to follow given the growing evidence supporting a role for the microbiota in the aging process (Heintz and Mair, 2014).

Altogether, our results reveal that *L.p.^WJL^* is overall beneficial for *Drosophila melanogaster*; the presence of these bacteria is profitable during larval life and does not harm the adult flies. Indeed, upon nutritional challenge, *L.p.^WJL^* allows the earlier emergence of fit and fertile adults and, in certain conditions, it even increases the lifespan of males. This *Lactobacillus* strain thus represents an advantageous partner for the fly, and taken together our results support the idea that commensal bacteria might be one of the factors contributing to the ecological success of *Drosophila*.
